# Ischemic and Oxidative Damage to the Hypothalamus May Be Responsible for Heat Stroke

**DOI:** 10.2174/1570159X11311020001

**Published:** 2013-03

**Authors:** Sheng-Hsien Chen, Mao-Tsun Lin, Ching-Ping Chang

**Affiliations:** a Department of Obstetrics and Gynecology, Chi Mei Medical Center, Tainan, Taiwan; b Department of Biotechnology, Southern Taiwan University of Science and Technology, Tainan, Taiwan; c Department of Medical Research, Chi Mei Medical Center, Tainan, Taiwan

**Keywords:** Hypothalamus, heatstroke, hypotension, ischemia, hypoxia, oxidative stress, cytokines, inflammation, antioxidants, pharmacology.

## Abstract

The hypothalamus may be involved in regulating homeostasis, motivation, and emotional behavior by controlling autonomic and endocrine activity. The hypothalamus communicates input from the thalamus to the pituitary gland, reticular activating substance, limbic system, and neocortex. This allows the output of pituitary hormones to respond to changes in autonomic nervous system activity. Environmental heat stress increases cutaneous blood flow and metabolism, and progressively decreases splanchnic blood flow. Severe heat exposure also decreases mean arterial pressure (MAP), increases intracranial pressure (ICP), and decreases cerebral perfusion pressure (CPP = MAP – ICP), all of which lead to cerebral ischemia and hypoxia. Compared with normothermic controls, rodents with heatstroke have higher hypothalamic values of cellular ischemia (e.g., glutamate and lactate-to-pyruvate ratio) and damage (e.g., glycerol) markers, pro-oxidant enzymes (e.g., lipid peroxidation and glutathione oxidation), proinflammatory cytokines (e.g., interleukin-1β and tumor necrosis factor-α), inducible nitric oxide synthase-dependent nitric oxide, and an indicator for the accumulation of polymorphonuclear leukocytes (e.g., myeloperoxidase activity), as well as neuronal damage (e.g., apoptosis, necrosis, and autophagy) after heatstroke. Hypothalamic values of antioxidant defenses (e.g., glutathione peroxidase and glutathione reductase), however, are lower. The ischemic, hypoxic, and oxidative damage to the hypothalamus during heatstroke may cause multiple organ dysfunction or failure through hypothalamic-pituitary-adrenal axis mechanisms. Finding the link between the signaling and heatstroke-induced hypothalamic oxidative and ischemic damage might allow us to clinically attenuate heatstroke. In particular, free radical scavengers, heat shock protein-70 inducers, hypervolemic hemodilution, inducible nitric oxide synthase inhibitors, progenitor stem cells, flutamide, estrogen, interleukin-1 receptor antagonists, glucocorticoid, activated protein C, and baicalin mitigate preclinical heatstroke levels.

## 1 INTRODUCTION

Heatstroke is a form of hyperthermia associated with a systemic inflammatory response that leads to multiorgan dysfunction syndrome (MODS), in which central nervous system (CNS) disorders (e.g., delirium, convulsion, and coma) predominate [1].

Passive heatstroke is experienced primarily by the very young or the elderly during annual heat waves [2]. Pre-existing conditions such as diabetes, alcoholism, or drug use may increase the incidence of classic heatstroke.

Recent epidemiological studies of heatstroke [3, 4] show that the goal of clinical therapies is to normalize body (core and skin) temperature and brain function as rapidly as possible, but ~30% of heatstroke survivors experience disability as well as neurological dysfunction. MODS continues to manifest in heatstroke patients after whole-body cooling [3-5]. Accumulated evidence indicates that, in an anesthetized-rodent model, brain ischemia, inflammation, and neuronal damage, rather than body hyperthermia, are the main causes of heatstroke [6-9]. Heatstroke-induced deaths are increasing with global warming and with a world-wide increase in the frequency and intensity of heat waves [10]. In terms of the clinical burden on the society, heatstroke-induced injury is the third largest killer in the world after cardiovascular disease and traumatic insults to the CNS [10, 11]. Because ischemic stroke and heatstroke are analogous (the basic mechanism of heatstroke is ischemic injury), recent therapeutic modalities tried in ischemic stroke may also be effective for heatstroke. For example, human clinical studies [12] report that hyperbaric oxygen therapy may be beneficial for treating chronic vascular disease and when using a cardiopulmonary bypass. This is confirmed by findings [13] showing that hyperbaric oxygen therapy has been used to successfully treat the MODS of a patient with heatstroke, and that recombinant human activated protein C (APC) improves outcomes in humans with heatstroke [14]. Dantrolene sodium, however, was not effective in a double-blind, randomized study [15].

The hypothalamus is believed to be involved in regulating homeostasis, motivation, and emotional behavior. These functions are mediated through hypothalamic control of autonomic and endocrine activity [16, 17]. The hypothalamus receives input from the thalamus, reticular activating substance, limbic system, eyes, and neocortex; it then transmits this input to the pituitary gland. This allows the output of pituitary hormones to respond to changes in the autonomic nervous system activity and to the needs of temperature regulation, water balance, and energy requirements [16].

Heat exposure is a stimulus that triggers biological stress reactions [18]. An increase in c-fos mRNA and protein in different brain regions, including the hypothalamus, the thalamus, and the amygdala, occurs in heat-exposed rats [19]. Large releases of hippocampal norepinephrine [20], hypothalamic dopamine and serotonin [21], and striatal glutamate [22] have also been reported. The hypothalamo-pituitary-adrenocortical (HPA) axis is also mobilized, as suggested by the increase in c-fos-positive cells [23] and c-fos mRNA content [24] in the hypothalamic paraventricular nucleus (PVN), as well as by the increase in blood adrenocorticotrophic-hormone (ACTH) and corticosterone concentrations [25, 26]. Furthermore, the HPA axis mobilization is accompanied by activation of the sympathetic nervous system [27, 28]. The hypothalamic PVN has been described as the “autonomic master controller” [17]. It coordinates critical physiological responses by controlling the HPA axis. Decreased heat tolerance has been associated with HPA axis impairment [29]. More than half a century ago, thermal injury to the thermoregulatory centers of the hypothalamus was hypothesized to be the primary mechanism of mortality [30].

This review will provide current evidence and discuss the hypothesis that ischemic and oxidative damage to the hypothalamus is responsible for the occurrence of MODS in heatstroke *via *the mechanisms of the HPA axis.

## 2 DEFINITIONS OF CLASSIC AND EXERTIONAL HEATSTROKE

Heatstroke can be caused by severe heat exposure (classic heatstroke) or strenuous work (exertional heatstroke). It is characterized by a myriad of inflammation, coagulation, and tissue injuries [1]. Heatstroke is a medical emergency that requires prompt intervention. The classic syndrome of heatstroke is a manifestation of critical hyperthermia with neurological decompensation that results in delirium, convulsion, or coma [1]. Two major findings—hyperthermia and CNS dysfunction (such as delirium, convulsion, or coma)—must be present for a diagnosis of heatstroke [1]. Because it is characterized by a wide spectrum of metabolic alterations and multiple organ failure, heatstroke shares similarities with other systemic inflammatory response syndromes [1, 31].

Classic heatstroke is caused by passive exposure to a hot environment in immunocompromised and aging populations. Pre-existing conditions, including mental illness, alcoholism, diabetes [31], or drug use, can predispose individuals to classic heatstroke. Athletes [32], manual laborers who work outdoors, and military personnel may increase the incidence of exertional heatstroke. The high death toll during heat waves may be related to culture, urban structure, the duration of heat exposure, a lack of heat acclimatization, concurrent infections, and genetic factors [2].

## 3 THE EFFECT ON COAGULATION, AND CONCEPTS OF EXERTIONAL HEATSTROKE WOULD HAVE BEEN PERTINENT

Hyperthermia due to passive heat exposure or to exercise may facilitate the leakage of endotoxin from the intestine to the systemic circulation, as well as the movement of interleukin (IL)-1 or IL-6 proteins from the muscles to the systemic circulation [1]. The result is an excessive activation of leukocytes and endothelial cells, manifested by the release of proinflammatory and anti-inflammatory cytokines, upregulation of cell-surface adhesion molecules, and shedding of soluble cell-surface adhesion molecules, as well as the activation of coagulation (with decreased levels of proteins C and S and antithrombin III) and the inhibition of fibrinolysis. The inflammatory and coagulation responses to heatstroke, together with direct cytotoxic effects of heat, injure vascular endothelium and cause microthrombosis.

Intense exercise suppresses anti-lipopolysaccharide (anti-LPS) antibodies (ABS) and T-helper cell 1 (Th1) immunity, and promotes LPS translocation and T-helper cell 2 (Th2) immunity. The suppression of anti-LPS ABS facilitates the accumulation of LPS in the circulation, which induces an increase in inflammatory and pyrogenic cytokines [32]. The suppression of Th1 and promotion of Th2 immunity, along with exercise-induced muscle tissue damage, also induces the synthesis and release of inflammatory and pyrogenic cytokines. These cytokines promote pyrogenesis, systemic inflammation, increased vascular permeability, and hypotension, which contribute to the occurrence of exertional heatstroke [32].

## 4 ANIMAL MODELS

Many thermoregulatory, hemodynamic, and tissue-injury complications are associated with heatstroke [1, 31]. However, in humans, a large variability in these complications may be due to several factors, including (i) differences in the time of clinical presentation, (ii) pre-clinical therapy, and (iii) individual differences in heat tolerance. It must be stressed that most of the observations made when studying the pathogenesis of heatstroke in humans have occurred during the hours or days after the onset of heatstroke. Relatively little information is available about the pathophysiological changes that occur right at the onset of heatstroke in humans [33].

In one animal study [8], heatstroke was induced by exposing restrained, unanesthetized rabbits to a high ambient temperature of 40°C. The occurrence of hyperthermia (43.5 ± 0.13°C) and CNS dysfunction (such as convulsions followed by coma) was taken as the onset of heatstroke. At the onset of heatstroke, the comatose rabbits showed hyperthermia and a reduction in both MAP (67 ± 2 mmHg) and CPP (due to decreased MAP and increased ICP).

Based on a triad of factors (hyperthermia, CNS disorders, and a history of heat stress), anesthetized and unanesthetized rodents (rabbits, rats, and mice) all display a uniform response: Their reactions are similar to those of humans with heatstroke [6, 34].

## 5 OVERALL CHANGES OF DIFFERENT PHYSIOLOGICAL AND HISTOPATHOLOGIC PARAMETERS IN THE BRAIN AFTER HEATSTROKE

After the onset of heatstroke, the reduction of blood flow to the brain (because of arterial hypotension and intracranial hypertension) causes neuronal injury because the blood flow reduction leads to oxygen and nutrient deprivation as well as to the initiation of a cascade of secondary mechanisms [7, 34, 35] (Table **1**). Rodents with heatstroke also display necrosis, apoptosis [7, 36-38], and autophagy [39].

## 6 ISCHEMIC AND OXIDATIVE DAMAGE TO THE HYPOTHALAMUS IN HEATSTROKE

Septic shock, a systemic inflammatory syndrome, is caused primarily by Gram-negative bacteria, which erupt when endotoxin or other microbial products initially produce tumor necrosis factor-α (TNF-α), IL-1, IL-6, interferon-γ, leukemia inhibitory factors, migration inhibitory factor, platelet activating factors, and products of the complement and clotting cascades [30]. Accumulated evidence suggests that oxidative stress and sepsis lead to multiorgan dysfunction or failure [40-42]. Endotoxin-activated macrophages and leukocytes also release reactive oxygen and nitrogen species (ROS and RNS) including superoxide, hydrogen peroxide, hydroxyl radical, nitric oxide (NO), and peroxynitrite, which generate multiorgan injury. Heatstroke is characterized by excessive hyperthermia associated with systemic inflammatory responses, which leads to multiorgan dysfunction or failure [1, 32, 34]. It is likely that RNS and ROS also generate heatstroke syndromes.

Types of heatstroke that resemble septic shock [32] are proposed to be a form of hyperthermia (core temperature rising above 40°C) associated with a systemic inflammatory response that leads to multiorgan dysfunction in which brain disorders predominate [1, 32, 34]. Various serum molecules, including TNF-α, soluble intercellular adhesion molecule-1, and E-selectin, are pathophysiologically involved in systemic inflammatory response syndrome [43-45]. Myeloperoxidase activity, an index of accumulating polymorphonuclear cells is also described [46]. Indeed, after the onset of heatstroke, rodents display hyperthermia, hypotension, hypothalamic apoptosis and degeneration (Table **1**), and the upregulation of systemic inflammatory response molecules—serum TNF-α, soluble intercellular adhesion molecule-1, and E-selectin [9], myeloperoxidase activity, and proinflammatory cytokines—all of which together generate multiorgan dysfunction (Table **2**) [9, 47, 48].

## 7 CYTOKINES, COAGULATION, AND HEATSTROKE

According to Chatterjee *et al.* [49, 50], heat-treated mice display core body temperatures of > 40°C immediately after the termination of 1 h of heat stress (~41°C), and profound hypothermia at +4, +6, and +20 h after. At +4 h and +24 h after heat stress, levels of IL-1β, nitrite, TNF-α, inducible nitric oxide synthase (iNOS), and corticosterone are significantly higher in the heatstroke group than in the sham group. This was recently confirmed by Lin *et al.* [51]. In fact, many of the characteristics of heatstroke syndromes resemble those of sepsis [1]. Any of the responses observed during septic shock can be mimicked by systemic administration of TNF-α [52]. Indeed, we previously [35, 53, 54] showed that the overproduction of IL-1 and TNF-α in both the peripheral blood stream and the CNS (including the hypothalamus) occurs in the rat during heatstroke. This is associated with hypotension, cerebral ischemia and neuronal damage, and shortened survival time. Administration of corticosteroids or cytokine receptor antagonists before the initiation of heat stress significantly attenuates circulatory shock, cerebral ischemia, and damage [54]. Thus, it appears that the overproduction of these proinflammatory cytokines may be positively correlated with mortality in rodents with heatstroke. However, this contention is not consistent with the findings of Leon *et al.* [55, 56], who reported on mice exposed to an ambient temperature of ~39.5°C until a maximum core temperature of 42.7°C was attained. During their recovery, the mice had hypothermia (29.3 ± 0.4°C) and, after 24 h of recovery, a fever-like elevation (37.8 ± 0.3°C) accompanied by insignificant changes in the plasma levels of both TNF-α and macrophage inflammatory protein-1α. IL-1β, IL-6, and IL-10 were inversely correlated with core temperatures; maximal production was during hypothermia, and IL-6 was elevated at 24 h. Leon *et al*. [55, 56] also reported that IL-6 and TNF-α double-receptor knockout mice had higher mortality rates than did their wild-type controls after a heatstroke collapse. They recognized that “these immune modulators elicit protective actions *in vivo* that are probably time and tissue specific”. It should be stressed that inflammatory responses in the initial phase of tissue injury might be involved in aggravating tissue damage, whereas in a later stage, these inflammatory mediators might contribute to recovery or repair [57]. In our study [51], when serum cytokine levels were determined at a single time point (2.5 h after heat stress termination), there was no discernable fever-like elevation (~37°C) at 24 h. Leon *et al.* [55, 56] also reported that IL-6^–/–^ mice had higher mortality rates, which suggested that IL-6 may be somehow protective. The discrepancy of our results with those of Leon *et al. *can be explained by time specificity*.* This might be because in the initial phase (onset) of heat stroke, overproduction of IL-6 or TNF-α has a detrimental effect on tissue injury, but in the later stage (recovery), these proinflammatory cytokines protect against tissue injury. Thus, it appears that anti-inflammatory agent therapy should be given in the early stage but not in the recovery period. In addition, other studies [49-51] reported profound hypothermia after heat stress, but Leon *et al.* [55, 56] did not. The hypothermia can be explained by heat-induced hypothalamic neuronal apoptosis [6, 51, 58, 59].

## 8 NITRIC OXIDE SYNTHASE INHIBITORS IN HEATSTROKE

NO, a major modulator of brain injury after ischemic events [60], can be toxic or protective to the ischemic brain. For example, compared with littermate controls, neuronal NOS (nNOS)-deficient mice had 38% smaller infarcts during cerebral ischemia [61]. iNOS is also induced in reactive astrocytes and in infiltrating neutrophils after cerebral ischemia [62]. In contrast, endothelial NOS (eNOS)-deficient mice had decreased blood flow in the periphery of the ischemic brain region where NO-mediated excitotoxicity is most prevalent, which increased the infarct size [63]. The evidence proves that overproducing NO from eNOS protects brain tissue by maintaining regional cerebral blood flow, but that producing NO from nNOS or iNOS leads to neurotoxicity.

In the rat, intestinal mucosal permeability to endotoxin increases during heatstroke [64]. This alteration increasingly produces proinflammatory cytokines that induce the overproduction of NO [65]. Inflammatory activation can interfere with cerebrovascular function, thereby precipitating heatstroke reactions [66]. Additionally, plasma or brain NO levels increase in patients [67] and rats [68-71] with heatstroke; treatment with L-NAME (N^ω^-nitro-L-arginine methyl ester), a non-selective eNOS inhibitor, at any time has proved not to prevent or reverse heatstroke in rats [71]. However, treatment with the iNOS inhibitor aminoguanidine [70], and with the nNOS inhibitor 7-nitroindazole (7-NI) [72], were beneficial for attenuating cerebrovascular dysfunction in rats with heatstroke. Hence, iNOS-dependent or nNOS-dependent NO formation in the brain appears to exacerbate neuronal ischemic injury during heatstroke or other ischemic injury.

## 9 FREE RADICAL SCAVENGERS AND HEATSTROKE

Heatstroke is associated with cerebral ischemia and higher levels of interleukin-1β, dopamine, and glutamate in the brain [72-75]. These factors are known to increase free radical production [76, 77]. Indeed, at the onset of heatstroke, the extent of lipid peroxidation and the rate of superoxide (O2^-^) generation in the brain are significantly elevated [78]; however, the values of superoxide dismutase (SOD) and catalase in the brain significantly decrease. These findings are consistent with several studies [79, 80] which show that ROS are involved in the ischemic brain injury of many neurological disorders. At the onset of heatstroke, many endogenous antioxidative defenses are likely to be perturbed as a result of the overproduction of ROS by cytosolic pro-oxidant enzymes and by the inactivation of detoxification systems, consumption of antioxidants, and failure to adequately replenish antioxidants in heat-injured (due to hyperthermia) and ischemic (due to ensuing hypotension) brain tissue by mitochondria. These ROS are directly involved in oxidative damage with macromolecules in ischemic tissue, which leads to cell death [76]. An increased level of dopamine, serotonin, glutamine, cytokines [66], or iNOS-dependent NO [70] is cerebral ischemia-associated in a rat heatstroke model. All these neuro-transmitters and cytokines may increasingly release ROS and result in brain ischemia and damage [81-83].

As mentioned above, cerebral ischemia and injury are associated with increased free radicals, increased lipid peroxidation, and decreased enzymatic antioxidant defenses in the brain of rats with heatstroke. This raises the possibility that pretreatment with free radical scavengers can protect against heatstroke-induced cerebral ischemia and damage. Indeed, pretreatment with α-tocopherol or mannitol 30 min before the onset of heat exposure significantly attenuates heatstroke-induced hypotension, cerebral ischemia and damage, increased brain levels of free radicals and lipid peroxidation, and increased plasma levels of proinflammatory cytokines [84]. Additionally, magnolol also significantly attenuates heatstroke-induced cerebral ischemia and damage and increased levels of free radicals and lipid peroxidation in the brain [85]. Sheng Mai San, a traditional Chinese herbal medicine, is routinely used to treat coronary heart disease [86, 87]. It effectively suppresses the formation of thiobarbituric acid reactive substances (TBARS) during reperfusion after ischemia, which indicates that Sheng Mai San attenuates oxidative damage to the brain [88]. Sheng Mai San attenuates the increase of free radicals and lipid peroxidation in the brain of rats with heatstroke [89].

## 10 HEAT SHOCK PROTEIN 72 (OR 70) PRECONDITIONING

Heat shock protein (HSP) 72 expression increases the tolerance of cerebral neurons to ischemic change [90] and protects neurons against subsequent severe heat shock (47°C) [91]. Moreover, heat shock preconditioning protects against heat-induced cerebral ischemia and damage by inducing HSP72 [92], and it may protect against heatstroke-induced cerebral ischemia by reducing oxidative stress and energy depletion [78]. Other evidence [93-95] shows that HSP72 can be detected in the skeletal muscle, heart, liver, and adrenal glands in rats that have undergone endurance training for 7-12 weeks. In addition, progressive exercise preconditioning in animal models of heatstroke induces HSP72 and attenuates oxidative damage as well as cerebral ischemia and damage [96]. When transgenic mice heterozygous for a porcine HSP70i gene ([+]HSP72), transgene-negative littermate controls ([–]HSP72), and normal Institute of Cancer Research-strain mice were subjected to heat stress (40°C) [97], HSP72 overexpression in multiple organs of the [+]HSP72 mice improved heatstroke survival by reducing cerebral ischemia and damage. This finding suggested that the protective effects of HSP72 overexpression might be related to the attenuation of oxidative damage to the brain [98]. Furthermore, giving heat-stressed mice L-arginine increased HSP72 expression and protected them from heat-induced death [49]. Glutamine also increased HSP72 expression and reduced cytokine release, gut permeability, and mortality in a rat heatstroke model [99]. It is likely that L-arginine or glutamine attenuates oxidative stress during heatstroke by inducing HSP72. Preinducing HSP70 overexpression using geranylgeranylacetone [100] or hypobaric hypoxia [101, 102] also attenuated heat-induced activated inflammation and multiple organ dysfunction in rats.

## 11 HYPERVOLEMIC HEMODILUTION

Hypervolemic hemodilution has been used to treat animals with acute focal cerebral ischemia [103]: it increases blood flow by reducing the viscosity and oxygen content of the blood caused by lower levels of hematocrit and fibrinogen [104]. Indeed, heatstroke-induced arterial hypotension, intracranial hypertension, cerebral hypoperfusion, cerebral ischemia, and neuronal damage can be significantly reduced by hypervolemic hemodilution (induced by systemically administering 10% human albumin [105], hypertonic saline (3%) [106], or small-volume resuscitation [107] before or after the onset of heatstroke). The beneficial effects of hypervolemic hemodilution are related to attenuating oxidative damage during heatstroke.

## 12 RECOMBINANT HUMAN ACTIVATED PROTEIN C THERAPY IN HEATSTROKE

Recombinant human APC is recommended for patients at a high risk of death (septic shock, sepsis-induced acute respiratory distress syndrome, Acute Physiology and Chronic Evaluation II score ≥ 25, and sepsis-induced multiple organ failure) who have no absolute contraindication related to a risk of bleeding or relative contraindication outweighing the potential benefit [108]. We previously [109, 110] provided data showing that systemic delivery of recombinant human APC at the onset of heatstroke in rats improved survival by ameliorating systemic inflammation, the hypercoagulable state, tissue ischemia, and injury in multiple organs of rats with heatstroke. These experimental animal results can be supported by the complete recovery of 2 patients with heatstroke who were treated with recombinant human APC [14]. In addition, in mice, APC therapy significantly decreased hypothalamic levels of heat-induced apoptosis and TNF-α, cellular ischemia (e.g., glutamate, lactate-to-pyruvate ratio, nitrite, and dihydroxybenzoic acid (DHBA)), and damage (e.g., glycerol) markers in the hypothalamus during heatstroke. These findings suggest that human recombinant APC mitigates heatstroke by restoring normal hypothalamic and thermoregulatory functions. However, a more recent report [111] showed that recombinant APC given to baboons with heatstroke provided cytoprotection, but had no effect on heatstroke-induced coagulation activation and fibrin formation.

## 13 COOLING THERAPY IN HEATSTROKE

Whole-body cooling is the current therapy of choice for heatstroke because no pharmacologic agent is available [11]. We showed [112] that whole-body cooling protected against heatstroke by reducing hypoxemia, lactacidemia, acidosis, hypotension, cerebral ischemia, and neuronal damage. In addition, we used the hypothermic retrograde jugular venous flush [78, 113, 114] to cool the brain in the rat and to provide better protection than peripheral cold saline perfusion during heatstroke. Compared with the normothermic controls, the 36°C saline-treated rats had higher brain temperatures, intracranial pressures, serum and hypothalamic NO metabolite, TNF-α, DHBA, and hypothalamic iNOS immunoreactivity. In contrast, MAP and CPP values, hypothalamic levels of local blood flow, and the partial pressure of oxygen were all significantly lower during heatstroke. Brain cooling significantly reduced the ischemic and oxidative damage to the hypothalamus during heatstroke [35]. These findings suggest that whole-body or brain cooling may resuscitate patients with heatstroke by decreasing the overproduction of RNS, TNF-α, and ROS that occurs in the hypothalamus and during cerebrovascular dysfunction.

## 14 MELATONIN THERAPY IN HEATSTROKE

Melatonin is beneficial for attenuating MODS in septic shock. Heatstroke resembles septic shock in many ways. We evaluated the effect of melatonin, a versatile molecule synthesized in the pineal gland and in many organs, in heatstroke and showed that melatonin significantly (i) attenuated hyperthermia, hypotension, and hypothalamic ischemia and hypoxia; (ii) reduced the plasma index of the toxic oxidizing radicals like NO metabolites and hydroxyl radicals; (iii) diminished the plasma index of hepatic and renal dysfunction, such as creatinine, blood urea nitrogen, alanine aminotransferase, aspartate aminotransferase, alkaline phosphatase, and lactate dehydrogenase; (iv) attenuated plasma systemic inflammation response molecules such as soluble intracellular and lesion molecule-1, E-selectin, TNF-α, IL-1β, and IL-6; (v) promoted plasma levels of IL-10; (vi) reduced myeloperoxidase activity in the lung, an index of infiltration of polymorphonuclear neutrophils; and (vii) quadrupled survival time compared with the heatstroke-alone group. Thus, melatonin might be a novel agent for treating animals or patients in the early stage of heatstroke [115, 116].

## 15 ESTROGEN AND FLUTAMIDE THERAPY IN HEATSTROKE

Accumulated evidence indicates that estradiol influences the severity of injuries associated with cerebrovascular stroke. For example, an ovariectomy in female rats eliminates the endogenous protection of estrogen after cerebral ischemia [117-119]. In addition, plasma estrogen levels are inversely related to ischemic stroke damage in female rats [120]. Estrogen replacement in adult ovariectomized rats significantly restored neuroprotection to a level similar to that in intact animals [117-119]. After a trauma and hemorrhage [121, 122], estrogen regulates the release of proinflammatory and anti-inflammatory cytokines. Compared with normothermic controls, vehicle-treated ovariectomized rats had higher levels of serum TNF-α, which were reduced by conjugated estrogen therapy. In contrast, serum levels of the anti-inflammatory cytokine IL-10 in these groups were significantly elevated by conjugated estrogens during heatstroke [123]. Furthermore, heatstroke-induced hyper-thermia, hypotension, and intracranial hypertension, as well as cerebral hypoperfusion, hypoxia, and ischemia were all significantly attenuated by conjugated estrogen therapy in ovariectomized rats. This demonstrates that estrogen replacement may improve survival by reducing inflammatory responses and cerebrovascular dysfunction during heatstroke.

Flutamide, a non-steroidal androgen receptor antagonist, has been used as an adjunct after trauma (e.g., hemorrhage) for restoring immune and cardiovascular function and for decreasing the mortality from subsequent sepsis [124, 125]. We evaluated the effects of flutamide in mice with heatstroke [51] and showed that flutamide significantly (i) attenuated hypothermia, (ii) reduced the number of apoptotic cells in the hypothalamus, the spleen, the liver, and the kidney, (iii) attenuated the plasma index of toxic oxidizing radicals (nitric oxide metabolites and hydroxyl radicals), (iv) diminished the plasma index of the organ injury index (LDH), (v) attenuated plasma systemic inflammatory response molecules (e.g., TNF-α and IL-6), (vi) reduced the index of infiltration of polymorphonuclear neutrophils in the lung (myeloperoxidase activity), and (vii) allowed three times the fractional survival during heatstroke compared with vehicle. Thus, flutamide appears to be a novel agent for treating early-stage heatstroke.

## 16 HUMAN UMBILICAL CORD BLOOD CELLS (HUCBCS) AND GRANULOCYTE-COLONY STIMULATING FACTOR (G-CSF) IN HEATSTROKE

HUCBCs are rich in hematopoietic stem cells [124]. Two percent of HUCBCs are stem cells capable of reconstituting blood lineages. When HUCBCs are administered *via *the tail vein, surviving HUCBCs can be identified in the injured hemisphere [125, 126]. The behavioral dysfunctions produced by stroke [127, 128], traumatic brain injury [129], and spinal cord injury [130] are significantly improved by the intravenous delivery of HUCBCs. We also demonstrated [131, 132] that the intravenous delivery of HUCBCs resuscitated rats with heatstroke by reducing circulatory shock and cerebral ischemia.

G-CSF, a polypeptide growth factor, stimulated the proliferation, survival, and maturation of the neutrophilic granulocyte lineage [132]. G-CSF was beneficial for decreasing the mortality rate, infarction volume, and neurological deficits in rats after cerebral ischemia [133]. G-CSF reduced heatstroke-induced hyperthermia, hypotension, increased serum levels of systemic inflammatory response syndrome, and increased hypothalamic apoptosis and neuronal damage scores [134]. Thus, our results reveal a potential for G-CSF used as a prophylactic agent for heatstroke.

## 17 IL-1 RECEPTOR ANTAGONIST IN HEAT-STROKE

The proinflammatory cytokine IL-1 is an important mediator of heatstroke. Humans with heatstroke have higher serum levels of IL-1 [135]. In a rabbit model, IL-1 levels in both serum and the brain increased after the onset of heatstroke [136]. Systemic administration of IL-1 receptor antagonist significantly reduced hyperthermia as well as cerebrovascular dysfunctions that occurred during heatstroke in rabbits [6, 136]. The data indicate that IL-1 receptor antagonist therapy may restore tissue blood flow and homeostatic function, and limit multiorgan dysfunction and death in heatstroke.

## 18 BAICALIN THERAPY IN HEATSTROKE

Baicalin (7-D-glucuronic acid-5,6-dihydroxy flavone), a flavonoid compound derived from the root of *Scutellaria baicalensis* Georgi, has been widely used in traditional Chinese herbal medicine to treat upper respiratory and gastrointestinal tract infections [137]. Baicalin is an antioxidant [138]. Baicalin may reduce fever by inhibiting both N-methyl-D-aspartate receptor-dependent hydroxyl radical pathways in the hypothalamus and TNF-α overproduction during endotoxin-induced fever [139]. In a rat model of hypoxic-ischemic brain damage, baicalin may prevent neuronal damage by downregulating apoptosis [140]. Indeed, administration of baicalin before heat exposure significantly attenuated hyperthermia, intracranial hypertension, and increased levels of NO, glutamate, glycerol, lactate-to-pyruvate ratio, and DHBA in the hypothalamus that occur in rats during heatstroke [141]. Baicalin pretreatment increased serum and hypothalamic levels of IL-1β and TNF-α, and reduced heatstroke-induced renal and hepatic dysfunction. In contrast, baicalin significantly increased both the serum and hypothalamic levels of IL-10 during heatstroke. These results strongly suggest that baicalin improves the outcome of heatstroke by reducing activated inflammation, multiple organ dysfunction, and ischemic and oxidative damage to the hypothalamus.

## 19 THE PATHOGENIC SEQUENCE IN THE HYPOTHALAMUS DURING HEATSTROKE

Fig. (**1**) depicts the proposed pathogenic sequence in the hypothalamus during heatstroke. Environmental heat stress increases cutaneous blood flow and metabolism, and progressively decreases splanchnic blood flow. Severe heat stress also decreases MAP, increases ICP, and results in decreased CPP, which leads to cerebral ischemia and hypoxia. Compared with normothermic controls, rodents with heatstroke have more hypothalamic markers of cellular ischemia (e.g., glutamate, lactate-to-pyruvate ratio) and damage (e.g., glycerol, LDH), and higher hypothalamic values of pro-oxidant enzymes (e.g., lipid peroxidation, glutathione oxidation), proinflammatory cytokines (e.g., IL-1β, TNF-α), iNOS-dependent NO, and myeloperoxidase activity, as well as more neuronal damage (apoptosis, necrosis, autophagy) after heatstroke. In contrast, rodents with heatstroke have antioxidants in the hypothalamus (e.g., glutathione peroxidase, glutathione reductase superoxide bismuthal). The ischemic, hypoxic, and oxidative damage to the hypothalamus during heatstroke may lead to MODS.

Studies [31, 36, 140, 141] have detected hypothalamic ischemic, hypoxic, and oxidative injury with a core body temperature of about 43°C. Ischemic, hypoxic, and oxidative damage to the hypothalamus during heatstroke might be responsible for the occurrence of MODS through hypothalamic-pituitary-adrenal axis mechanisms [142, 143].

## 20 CONCLUSIONS

The major heatstroke syndromes include hyperthermia, hypotension, intracranial hypertension, and hypothalamic ischemia and hypoxia (Table **1**). After the onset of heat-stroke, neuronal necrosis, apoptosis, and autophagy also occur in the hypothalamus.

The major known immunoinflammatory mediators in the hypothalamus contain more cellular ischemia markers (e.g., glutamate, lactate-to-pyruvate ratio), more cellular damage markers (e.g., glycerol, LDH, dopamine, 5-hydroxytryptamine), more proinflammatory cytokines (e.g., TNF-α, IL-1β), more oxidized radicals (e.g., iNOS-dependent NO, DHBA), and more pro-oxidant enzymes (e.g., lipid peroxidation, glutathione oxidation) (Table **2**). In contrast, the hypothalamus of rodents with heatstroke contains fewer antioxidants (e.g., glutathione peroxidase, glutathione reductase, SOD).

Free radical scavengers, heat shock protein-70 preconditioning, hypervolemic hemodilution, iNOS inhibitors, melatonin, progenitor stem cells (CD34^+^ cells), flutamide, estrogen, IL-1 receptor antagonist, glucocorticoids, APC, body cooling, and baicalin are all beneficial for treating pre-clinical levels of heatstroke (Table **3**).

Taking these findings together, we conclude that ischemic, hypoxic, and oxidative damage to the hypothalamus is involved in the pathogenesis of heatstroke. To find the signaling links for the ischemic and oxidative damage will be challenging. Once we know what they are, perhaps we will be able to clinically quench heatstroke.

## Figures and Tables

**Fig. (1):**
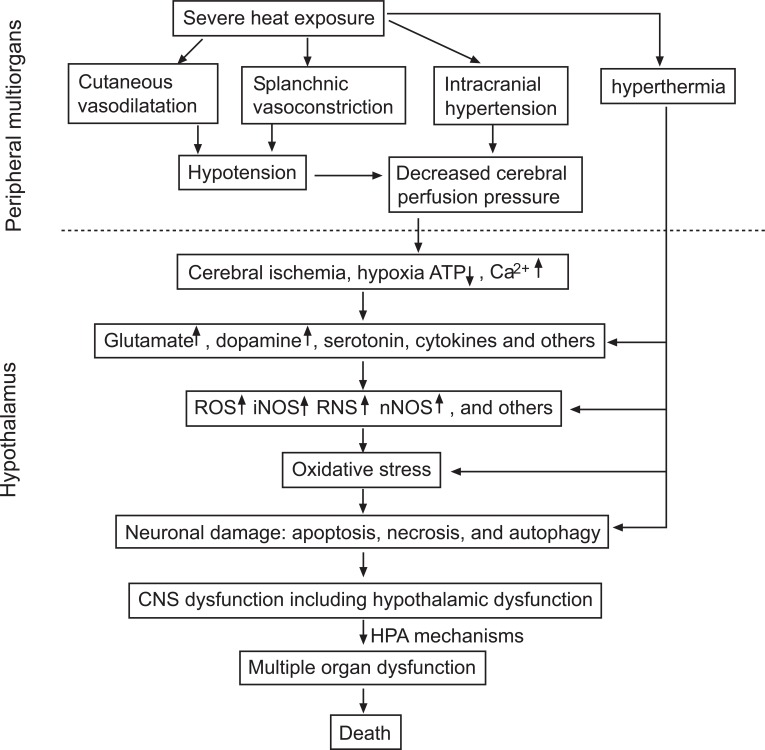
A flowchart of the proposed pathogenic sequence in the hypothalamus during heatstroke. Environmental heat stress increases cutaneous blood flow and metabolism, and progressively decreases splanchnic blood flow. Severe heat exposure also decreases mean arterial pressure, increases intracranial pressure, and results in decreased cerebral perfusion pressure, which leads to cerebral ischemia and hypoxia. Compared with normothermic controls, rodents with heatstroke have more hypothalamic markers of cellular ischemia and damage, pro-oxidant enzymes, and higher hypothalamic values of proinflammatory cytokines, iNOS-dependent NO, and an indicator of the accumulation of polymorphonuclear leukocytes, as well as more neuronal damage after heatstroke. In contrast, they have fewer antioxidants in the hypothalamus. The ischemic, hypoxic, and oxidative damage to the hypothalamus during heatstroke may lead to MODS.

**Table 1.** Overall Changes of Different Physiological and Histopathologic Parameter in the Brain (or Hypothalamus) after Heatstroke

Parameters	Consequences [References]
1. Physiological parameters:	
a) Body core temperature (Tco)	increased [7, 34, 35]
b) Mean arterial pressure (MAP)	decreased [7, 34, 35]
c) Intracranial pressure (ICP)	increased [7, 34, 35]
d) Cerebral perfusion pressure (CPP = MAP - ICP)	decreased [7, 34, 35]
e) Cerebral blood flow (CBF)	decreased [7, 34, 35]
f) Brain PO_2_	decreased [7, 34, 35]
2. Histopathological parameters	
a) Degeneration (Necrosis)	increased [7, 36]
b) Apoptosis	increased [37, 39, 47, 48, 97]
c) Autophagy	increased [39]

**Table 2.** Overall Changes of Different Components in the Brain (or Hypothalamus) after Heatstroke

Parameters	Consequences	References
1. Systemic inflammatory response syndrome molecules:		[36-38]
a) Tumor necrosis factor-α (TNF-α)	Increased	
b) Interleukin-6 (IL-6)	Increased	
c) Interleukin-1β (IL-1β)	Increased	
d) Myeloperoxidase	Increased	
e) E-selectin	Increased	
f) ICAM-1 (intercellular adhesion molecule-1)	Increased	
2. Cellular ischemia markers:		[7, 34, 36]
a) Glutamate	Increased	
b) Lactate-to-pyruvate ratio	Increased	
3. Cellular damage or organ injury markers:		[73, 115]
a) Glycerol	Increased	[73, 115]
b) Lactate dehydrogenase (LDH)	Increased	[73]
c) Dopamine, 5-hydroxytryptamine	Increased	[73]
4. Glutathione system		[78, 79]
a) GSH	Decreased	[78, 79]
b) GSSG	Increased	[78, 79]
c) GSSG/GSH	Increased	[78, 79]
5. Oxidative stress markers		[61]
a) NOx (nitric oxide metabolites)	Increased	[70, 70]
b) DHBA (dihydroxybenzoic acid)	Increased	[78, 79]
6. Lipid peroxidation		[78, 79]
a) MDA (malondialdehyde)	Increased	[78, 79]
7. Antioxidant enzyme estimation		[78, 79]
a) SOD (superoxide dismutase)	Decreased	
b) GPx (glutathione peroxidase)	Decreased	
c) GR (glutathione reductase)	Decreased	

**Table 3.** Potential Therapeutic Approaches for Brain Oxidative Damage in Classic Heatstroke

	References
1. Free radical scavengers:	
a) α-tocopherol	[84]
b) Mannitol	[84]
c) Magnolol	[85]
d) Shengmai San	[89]
2. Heat shock protein 70 preconditioning	[96-102]
a) Heat shock preconditioning	[96-99]
b) Hypoxia preconditioning	[101, 102]
c) Exercise	[96]
d) Drugs	[99, 100]
3. Hypervolemic hemodilution	[22, 106, 107]
a) 3% NaCl	[106]
b) 10% Human albumin	[22]
c) Hydroxyethyl starch	[107]
4. Nitric oxide synthase inhibitors (inducible or neuronal)	[59, 61]
a) Aminoguanidine	[70]
b) 7-nitroindazole (7-NI)	[72]
5. Melatonin	[115, 116]
6. Progenitor stem cells	[130, 131]
7. Flutamide, estrogen	[51, 58, 59, 123]
8. Interleukin-1 (IL-1) receptor antagonist	[6, 136]
9. Glucocorticoids	[54]
10. Body cooling	[112-114]
11. Activated protein C (APC)	[109, 110]
12. Baicalin (a flavonoid)	[141]
